# Supplementary material for: ICU-acquired infections and mortality in community-acquired pneumonia–induced sepsis: Insights from a transcriptomic analysis
Source: PLoS One. 2026 Jun 5;21(6):e0349871. doi: 10.1371/journal.pone.0349871 (PMC13240890; doi:10.1371/journal.pone.0349871)
Supplement: Attachment — Submitted filename: Response to Reviewers.docx [file pone.0349871.s002.docx]

Dear Reviewers,

We are pleased to submit a revised version of our manuscript (Manuscript ID: PONE-D-25-66274) entitled *"* ***ICU-acquired infections drive higher mortality in community-acquired pneumonia–induced sepsis: insights from a transcriptomic analysis*** *"* for consideration. We would like to thank you for the time spent reviewing the manuscript and for your insightful comments and suggestions for improvements to the manuscript.

We have responded to the comments completely, as detailed in the point-by-point responses below. We hope that this revised manuscript is suitable for publication in *PLOS One*.

Please submit your revised manuscript by Apr 17 2026 11:59PM. If you will need more time than this to complete your revisions, please reply to this message or contact the journal office at [plosone@plos.org](mailto:plosone@plos.org). When you're ready to submit your revision, log on to https://www.editorialmanager.com/pone/ and select the 'Submissions Needing Revision' folder to locate your manuscript file.

Please include the following items when submitting your revised manuscript:

- A letter that responds to each point raised by the academic editor and reviewer(s). You should upload this letter as a separate file labeled **'Response to Reviewers'**.
- A marked-up copy of your manuscript that highlights changes made to the original version. You should upload this as a separate file labeled **'Revised Manuscript with Track Changes'**.
- An unmarked version of your revised paper without tracked changes. You should upload this as a separate file labeled '**Manuscript**'.

**ACADEMIC EDITOR:**

*Dear Authors,*

*Thank you for submitting your manuscript on ICU-acquired infections and 30-day mortality in pneumonia-induced sepsis, integrating transcriptomic analysis. This is an important and clinically meaningful topic. The attempt to connect clinical outcomes with gene expression patterns is a real strength of the study, and the comparison between community-acquired and hospital-acquired pneumonia adds valuable context. After carefully considering the reviewer’s comments and assessing the manuscript myself, I agree that the study addresses a relevant question. At the same time, there are several methodological issues that need to be addressed before the conclusions can be considered robust.*

1. *The central concern relates to confounding. Mortality comparisons are largely based on unadjusted survival analyses. Given the retrospective design and the relatively small number of ICU-acquired infection cases, it is difficult to determine whether ICU-acquired infection independently predicts mortality or simply reflects greater baseline severity. If severity variables such as SOFA, APACHE II, age, or comorbidities are available, incorporating them into a multivariable model would substantially strengthen the analysis. If this is not feasible, the limitations should be clearly acknowledged and the conclusions reframed accordingly. In particular, any language implying causality should be moderated.*

**Response to reviewer:** Thank you for this important comment. We agree that confounding and baseline illness severity are important considerations. However, the publicly available dataset does not include established severity indices such as SOFA or APACHE II, and the number of ICU-acquired infection cases was limited; therefore, multivariable adjustment was not feasible. We have clarified this point in the Methods and Limitations sections and moderated the interpretation accordingly. (Lines 128–131; Lines 359–371)

2. *The small number of events, especially within subgroup analyses, also warrants caution. Borderline p-values and subgroup findings should be interpreted carefully. Providing effect sizes with confidence intervals and explicitly discussing the limited statistical power would improve transparency.*

**Response to reviewer:** Thank you for this important comment. We agree that the small number of events warrants cautious interpretation. We have added effect sizes with 95% confidence intervals in the Results section and further emphasized the limited statistical power and cautious interpretation in the manuscript (Lines 176–187).

3. *Regarding the transcriptomic component, additional clarification would be helpful. Please describe how gene expression data were normalized, whether batch effects were assessed, and how the genes of interest were selected. Because multiple genes were evaluated, the issue of multiple comparisons should be addressed, either through appropriate correction or through clear justification. If the findings are exploratory, this should be stated explicitly.*

**Response to reviewer:** Thank you for this helpful suggestion. We have clarified the transcriptomic methodology in the Methods section. Specifically, we now describe the preprocessing of gene expression data (RMA background correction, quantile normalization, variance filtering, and batch-effect correction using SVA and ComBat), the literature-based selection of candidate genes, and the exploratory nature of the analysis. Because a limited number of candidate genes were evaluated, formal correction for multiple comparisons was not applied. These clarifications have been added to the Methods section (Lines 112–121).

4. *The definition of ICU-acquired infection also needs clearer description, including diagnostic criteria and timing relative to ICU admission. This information is essential for readers to interpret the findings appropriately.*

**Response to reviewer:** Thank you for this helpful comment. We have clarified the definition of ICU-acquired infection in the Methods section. ICU-acquired infection status was obtained from the clinical annotation variable provided in the GSE65682 dataset. In the original MARS cohort, ICU-acquired infections were defined as infections occurring more than 48 hours after ICU admission. This information has now been added to the Methods section to improve clarity (Lines 107–109)

5. *Overall, the study is potentially interesting and hypothesis-generating. With stronger statistical adjustment, clearer methodological reporting, and more cautious interpretation, it could make a meaningful contribution. I encourage you to revise the manuscript carefully and provide a detailed response to the reviewer’s comments.*

**Response to reviewer:** Thank you for the positive and constructive feedback. We have carefully revised the manuscript, clarified aspects of the methodology, and moderated the interpretation to emphasize the exploratory and hypothesis-generating nature of the study. These revisions have been incorporated throughout the manuscript.

**Journal Requirements:**

When submitting your revision, we need you to address these additional requirements.

***1.*** *Please ensure that your manuscript meets PLOS ONE's style requirements, including those for file naming. The PLOS ONE style templates can be found at*

<https://journals.plos.org/plosone/s/file?id=wjVg/PLOSOne_formatting_sample_main_body.pdf> and

[https://journals.plos.org/plosone/s/file?id=ba62/PLOSOne_formatting_sample_title_authors_affiliations.pdf](https://eur01.safelinks.protection.outlook.com/?url=https%3A%2F%2Ftrack.editorialmanager.com%2FCL0%2Fhttps%3A%252F%252Fjournals.plos.org%252Fplosone%252Fs%252Ffile%253Fid%3Dba62%252FPLOSOne_formatting_sample_title_authors_affiliations.pdf%2F1%2F010f019cb3122eff-f8e04cf7-b059-4a1d-89a7-ee4104a0d2bd-000000%2FZ6zpFmQCi-jrYxb6sihMFQhO69yp4aBdxXVyOJablzk%3D252&data=05%7C02%7Cs.almuntashiri%40uoh.edu.sa%7Ce905a5857cc74b5ea34e08de79099cc1%7C514faa198c844cef8ae92b9dbce933cd%7C1%7C0%7C639081280144922837%7CUnknown%7CTWFpbGZsb3d8eyJFbXB0eU1hcGkiOnRydWUsIlYiOiIwLjAuMDAwMCIsIlAiOiJXaW4zMiIsIkFOIjoiTWFpbCIsIldUIjoyfQ%3D%3D%7C0%7C%7C%7C&sdata=OK2vIRSmD2qnxHLzov0qB4FPI9NVqZtCggZOlBaQL7c%3D&reserved=0).

**Response:** We have carefully reviewed the manuscript and ensured that it complies with PLOS ONE style requirements

***2.*** *Thank you for stating the following financial disclosure: “This research is funded by Scientific Research Deanship at University of Ha’il- Saudi Arabia through project number BA-24 029.” Please state what role the funders took in the study.  If the funders had no role, please state: "The funders had no role in study design, data collection and analysis, decision to publish, or preparation of the manuscript." If this statement is not correct you must amend it as needed. Please include this amended Role of Funder statement in your cover letter; we will change the online submission form on your behalf.*

**Response:** The funders had no role in study design, data collection and analysis, decision to publish, or preparation of the manuscript. This statement has been added to both the manuscript and the cover letter. (Lines 420–421)

***3.*** *We note that the grant information you provided in the ‘Funding Information’ and ‘Financial Disclosure’ sections do not match. When you resubmit, please ensure that you provide the correct grant numbers for the awards you received for your study in the ‘Funding Information’ section.*

**Response:** Thank you for this comment. The grant information in the ‘Funding Information’ and ‘Financial Disclosure’ sections has now been reviewed and corrected to ensure consistency.

*4. We note that your Data Availability Statement is currently as follows: [All relevant data are within the manuscript and its Supporting Information files]. Please confirm at this time whether or not your submission contains all raw data required to replicate the results of your study. Authors must share the “minimal data set” for their submission. PLOS defines the minimal data set to consist of the data required to replicate all study findings reported in the article, as well as related metadata and methods (*[*https://journals.plos.org/plosone/s/data-availability#loc-minimal-data-set-definition*](https://journals.plos.org/plosone/s/data-availability#loc-minimal-data-set-definition)*). For example, authors should submit the following data: - The values behind the means, standard deviations and other measures reported; - The values used to build graphs; - The points extracted from images for analysis. Authors do not need to submit their entire data set if only a portion of the data was used in the reported study. If your submission does not contain these data, please either upload them as Supporting Information files or deposit them to a stable, public repository and provide us with the relevant URLs, DOIs, or accession numbers. For a list of recommended repositories, please see* [*https://journals.plos.org/plosone/s/recommended-repositories*](https://journals.plos.org/plosone/s/recommended-repositories)*. If there are ethical or legal restrictions on sharing a de-identified data set, please explain them in detail (e.g., data contain potentially sensitive information, data are owned by a third-party organization, etc.) and who has imposed them (e.g., an ethics committee). Please also provide contact information for a data access committee, ethics committee, or other institutional body to which data requests may be sent. If data are owned by a third party, please indicate how others may request data access.*

**Response:** Thank you for this important comment. We have updated the Data Availability Statement and provided the processed dataset used for the current analysis as Supporting Information to ensure that all results can be fully reproduced. (Lines 424–427)

*5. Your ethics statement should only appear in the Methods section of your manuscript. If your ethics statement is written in any section besides the Methods, please move it to the Methods section and delete it from any other section. Please ensure that your ethics statement is included in your manuscript, as the ethics statement entered into the online submission form will not be published alongside your manuscript.*

**Response:** The ethics statement has been moved to the Methods section, and it has been removed from the end sections of the manuscript as requested. (Lines 136–141)

**6.** *Please upload a copy of Figure 1 & 2, to which you refer in your text. If the figure is no longer to be included as part of the submission please remove all reference to it within the text.*

**Response:** Thank you for this comment. Figures 1 and 2 have been uploaded as separate files in accordance with PLOS ONE guidelines. Their titles and captions are included in the main manuscript immediately after their first mention in the text. (Lines 203–209 and Lines 242–256)

**7.** *Please include a copy of Table 1 & 2 which you refer to in your text.*

**Response:** Thank you for this comment. Tables 1 and 2 have been included in the main manuscript file and placed after their first citation in the text, following PLOS ONE formatting instructions. (Lines 151–155 and Lines 170–173)

***8.*** *If the reviewer comments include a recommendation to cite specific previously published works, please review and evaluate these publications to determine whether they are relevant and should be cited. There is no requirement to cite these works unless the editor has indicated otherwise.*

**Response:** Thank you for this comment. We carefully reviewed the reviewer comments and confirmed that no specific references were recommended for citation. Therefore, no additional citations were added.

**Reviewer 1:**

*This manuscript presents a secondary transcriptomic analysis of the GSE65682 dataset exploring the impact of ICU-acquired infections on 30-day mortality in pneumonia-induced sepsis, with subgroup analysis of CAP and HAP patients. The topic is clinically relevant, and the integration of outcome analysis with gene expression profiling is a strength of the study.*

1. *The conclusions occasionally imply mechanistic or causal relationships (e.g., immune dysregulation “may predispose” to ICU-acquired infections). Given the retrospective design and single time-point gene expression measurement at ICU admission, the findings should be framed more explicitly as associative rather than predictive or causal.*

**Response to reviewer:** Thank you for this helpful comment. We have revised the manuscript to moderate causal language and to frame the findings more clearly as associative rather than predictive. These changes have been implemented throughout the manuscript.

2. *Survival comparisons were performed using Kaplan–Meier analysis without multivariable adjustment. With only 20 ICU-acquired infection cases (11 in the CAP subgroup), confounding remains a concern. Please clarify whether severity indices (e.g., SOFA, APACHE II, if available in GSE65682) were examined, and discuss how the lack of adjustment may influence interpretation.*

**Response to reviewer:** Thank you for this important comment. We acknowledge that survival comparisons were based on Kaplan–Meier analysis without multivariable adjustment. However, the publicly available dataset does not include established severity indices such as SOFA or APACHE II, and the number of ICU-acquired infection cases was limited. We have clarified this point in the Methods and Limitations sections and moderated the interpretation accordingly. (Lines 129–131; Lines 359–371)

3. *Five genes were tested without correction for multiple comparisons. Even with a limited gene set, statistical correction (e.g., Bonferroni or FDR) should be considered or justified. The IL7R finding (p=0.04 overall; p=0.01 in CAP) may not remain significant after strict correction.*

**Response to reviewer:** Thank you for this important comment. The genes evaluated in this study were selected as biologically relevant candidate genes based on prior literature rather than through genome-wide screening. Therefore, the transcriptomic component of the analysis was considered exploratory. We have clarified this point in the Methods section to improve transparency. (Lines 117–121)

4. *The operational definition of ICU-acquired infection within the dataset is not clearly described. Please specify timing criteria, diagnostic definitions, and how these events were identified in the secondary dataset.*

**Response to reviewer:** Thank you for this important comment. We have clarified the operational definition of ICU-acquired infection in the Methods section. ICU-acquired infection status was obtained from the clinical annotation variable provided in the GSE65682 dataset. In the original MARS cohort, ICU-acquired infections were defined as infections occurring more than 48 hours after ICU admission. This clarification has been added to the Methods section to improve transparency and reproducibility. (Lines 107–109)

5. *The CAP subgroup with ICU-acquired infection includes only 11 patients. The borderline mortality difference (p=0.05) should be interpreted cautiously. A brief discussion of limited statistical power and potential instability of estimates would strengthen the manuscript.*

**Response to reviewer:** Thank you for this helpful comment. We agree that the CAP subgroup with ICU-acquired infection included a small number of patients. We have revised the Limitations section to clarify that the small sample size may limit statistical power and the stability of the estimates, and that the findings should therefore be interpreted cautiously. (Lines 359–371)

6. *Minor grammatical issues are present (e.g., “To evaluated the impact…” in the abstract).*

**Response to reviewer:** Thank you for this comment. The grammatical issues in the abstract have been corrected. (Line 25)

7. *Please clarify whether gene expression data were normalized and whether batch effects were assessed or corrected.*

**Response to reviewer:** Thank you for this comment. We have clarified in the Methods section that gene expression data preprocessing, including normalization and batch-effect correction, followed the procedures described in the original GSE65682 dataset. Briefly, the original dataset was processed using Robust Multi-array Average (RMA) normalization with assessment and correction of batch effects. (Lines 112–121)

8. *The discussion could be slightly condensed, particularly sections reiterating IL7R literature.*

**Response to reviewer:** Thank you for this helpful suggestion. We agree and have slightly condensed the IL7R-related discussion to improve clarity and reduce repetition. (Lines 287–301)

9. *Consider tempering claims regarding novelty of CAP vs HAP differential mortality unless supported by comprehensive literature evidence.*

**Response to reviewer:** Thank you for this helpful suggestion. We have carefully moderated the statement regarding novelty and revised the text to present this observation more cautiously. (Lines 270–286)

**Reviewer 2:**

*Thank you for the opportunity to review this manuscript examining the association between ICU acquired infections and 30 day mortality in pneumonia induced sepsis, together with related immune transcriptomic patterns. The topic is clinically important, and the integration of clinical outcomes with gene expression analysis is a meaningful and potentially valuable approach. The stratified analysis between community acquired pneumonia and hospital acquired pneumonia is particularly interesting and may have clinical implications. However, several methodological and interpretative concerns should be addressed before the manuscript can be considered suitable for publication in PLOS ONE.*

1. *The most important concern relates to confounding and the absence of multivariable analysis. Mortality comparisons rely primarily on Kaplan Meier curves and log rank testing, without adjustment for baseline severity, organ dysfunction, comorbidities, or other relevant ICU factors. Patients who develop ICU acquired infections are often more severely ill at baseline, and without proper adjustment it is not possible to determine whether ICU acquired infection independently predicts mortality or simply reflects greater illness severity. I strongly recommend performing multivariable Cox regression or logistic regression adjusting for available severity markers. Without such analysis, the conclusion that ICU acquired infections drive mortality should be softened to indicate association rather than causation.*

**Response to reviewer:** Thank you for this important comment. We agree that confounding and baseline illness severity are important considerations when interpreting the observed mortality differences. However, the publicly available dataset does not include established severity indices such as SOFA or APACHE II, and the number of ICU-acquired infection cases was limited, making multivariable adjustment not feasible. We have clarified this limitation in the Methods and Limitations sections and moderated the interpretation to emphasize association rather than causation. (Lines 129–131; Lines 359–371)

2. *The relatively small number of patients who developed ICU acquired infections also raises concerns regarding statistical power. Only twenty patients developed ICU acquired infections overall, and only eleven in the community acquired pneumonia subgroup. The reported p value of 0.05 in this subgroup is borderline and should be interpreted cautiously. The manuscript would benefit from reporting effect sizes with confidence intervals and explicitly acknowledging the limited precision of subgroup analyses.*

**Response to reviewer:** Thank you for this important comment. We agree that the small number of ICU-acquired infection cases, particularly within the CAP subgroup, limits statistical power and warrants cautious interpretation of borderline findings. We have now reported effect sizes with 95% confidence intervals and further emphasized the limited precision of subgroup analyses in the manuscript. (Lines 177–187)

3. *Regarding the transcriptomic analysis, the rationale for selecting the five immune related genes requires clarification. Since the dataset is genome wide, it is important to specify whether these genes were prespecified based on biological hypotheses or selected after exploratory screening. In addition, no correction for multiple testing is described. Given the number of genes and subgroup comparisons performed, adjustment for multiple comparisons would strengthen the robustness of the findings. While IL7R appears statistically significant, the remaining genes did not reach significance and should be clearly described as exploratory trends.*

**Response to reviewer:** Thank you for this helpful comment. We have clarified in the Methods section that the genes analyzed in this study were selected as biologically relevant candidate genes based on prior literature. We have also stated that the transcriptomic analysis was exploratory and clarified the interpretation of the gene expression findings in the Results section. (Lines 117–121)

4. *The biological discussion of IL7R is thoughtful and supported by prior literature. The link to immune suppression in sepsis is plausible and clinically relevant. However, translational implications should be presented more cautiously given the retrospective and hypothesis generating nature of the analysis. Functional validation and independent cohort replication would be necessary before proposing biomarker guided immune targeted strategies.*

**Response to reviewer:** Thank you for this helpful comment. We have revised the discussion to moderate the translational interpretation of the IL7R findings and emphasized the exploratory and hypothesis-generating nature of the analysis. The text now reflects the need for further functional validation and independent cohort replication before clinical application. (Lines 359–371)

5. *The manuscript would also benefit from clearer reporting of how ICU acquired infection was defined within the dataset. Diagnostic criteria, timing of onset, and confirmation methods should be described to improve transparency and reproducibility.*

**Response to reviewer:** Thank you for this helpful comment. We have clarified the definition of ICU-acquired infection in the Methods section. ICU-acquired infection status was obtained from the clinical annotation variable provided in the GSE65682 dataset. In the original MARS cohort, ICU-acquired infections were defined as infections occurring more than 48 hours after ICU admission. This clarification has been added to the Methods section to improve transparency and reproducibility. (Lines 107–109)

6. *The study has several strengths. The use of a publicly available dataset promotes transparency. The stratified analysis between community acquired pneumonia and hospital acquired pneumonia adds clinical nuance. The authors acknowledge several limitations. However, additional limitations should be emphasized, including lack of multivariable adjustment, small subgroup size, potential reverse causality, and absence of external validation.*

**Response to reviewer:** Thank you for this helpful comment. We agree and have revised the Limitations section to further emphasize several important considerations, including the lack of multivariable adjustment, the relatively small subgroup size, the possibility of reverse causality, and the absence of external validation. These points have now been clarified in the revised manuscript. (Lines 359–371)

7. *Minor issues include a grammatical error in the abstract where “To evaluated” should read “To evaluate,” and inconsistency in referring to 28 day versus 30 day mortality. These should be corrected for clarity.*

**Response to reviewer:** Thank you for this comment. The grammatical error in the Abstract has been corrected. In addition, the terminology has been standardized throughout the manuscript to consistently refer to 28-day mortality. (Lines 25; 31; 87; 176)

8. *In summary, the manuscript addresses an important clinical question and presents potentially interesting findings, particularly regarding IL7R expression in community acquired pneumonia induced sepsis patients who develop ICU acquired infections. However, the current analysis does not fully support the strength of the conclusions. I recommend major revision, with particular attention to multivariable adjustment, clarification of gene selection strategy, correction for multiple testing, clearer definition of ICU acquired infection, and moderation of causal language.*

**Response to reviewer:** Thank you for this thoughtful and constructive comment and for recognizing the potential relevance of our findings. We have carefully revised the manuscript to moderate causal language and strengthen the clarity of the interpretation. In addition, we clarified the gene selection strategy, the definition of ICU-acquired infection, and the exploratory nature of the transcriptomic analysis without multiple testing correction. The absence of multivariable adjustment has also been acknowledged in the limitations section.
