# Supplementary material for: ICU-acquired infections and mortality in community-acquired pneumonia–induced sepsis: Insights from a transcriptomic analysis
Source: PLoS One. 2026 Jun 5;21(6):e0349871. doi: 10.1371/journal.pone.0349871 (PMC13240890; doi:10.1371/journal.pone.0349871)
Supplement: Attachment — Submitted filename: Response_to_Reviewers_auresp_2.docx [file pone.0349871.s003.docx]

Dear Academic Editor and Reviewers,

We are pleased to submit a revised version of our manuscript (Manuscript ID: PONE-D-25-66274R1) entitled *"* ***ICU-acquired infections and mortality in community-acquired pneumonia–induced sepsis: insights from a transcriptomic analysis*** *"* for consideration. We would like to thank you for the time spent reviewing the manuscript and for your insightful comments and suggestions for improvements to the manuscript.

We have responded to the comments completely, as detailed in the point-by-point responses below. We hope that this revised manuscript is suitable for publication in *PLOS One*.

**Journal Requirements:**

*1. If the reviewer comments include a recommendation to cite specific previously published works, please review and evaluate these publications to determine whether they are relevant and should be cited. There is no requirement to cite these works unless the editor has indicated otherwise.*

**Response:** Thank you for this comment. We carefully reviewed the reviewer comments and confirmed that no specific references were recommended for citation. Therefore, no additional citations were added.

*2. Please review your reference list to ensure that it is complete and correct. If you have cited papers that have been retracted, please include the rationale for doing so in the manuscript text, or remove these references and replace them with relevant current references. Any changes to the reference list should be mentioned in the rebuttal letter that accompanies your revised manuscript. If you need to cite a retracted article, indicate the article’s retracted status in the References list and also include a citation and full reference for the retraction notice.*
**Response:** Thank you for this comment. We have carefully reviewed the reference list to ensure accuracy and completeness.

**Additional Editor Comments:** *Thank you for submitting the revised version of your manuscript. The reviewers appreciate the improvements made, particularly in terms of clarity and overall presentation, and the study addresses a clinically relevant question with a meaningful integration of clinical and transcriptomic data. To further strengthen the manuscript prior to acceptance, the interpretation of the findings could be slightly tempered to better reflect the exploratory nature of the analysis, particularly by avoiding causal language and ensuring that conclusions remain proportionate to the strength of the data. It would also be helpful to more explicitly elaborate on key limitations, including the absence of multivariable adjustment and the potential influence of confounding factors such as disease severity, comorbidities, or ICU-related interventions, as well as the relatively small sample size and associated uncertainty in the estimates. In addition, a clearer emphasis on the exploratory nature of the transcriptomic analysis, including a brief note on the absence of correction for multiple testing, would improve transparency. The discussion of IL7R may also benefit from being framed as hypothesis-generating, and the possibility of reverse causation could be acknowledged more explicitly. Overall, the manuscript is progressing well and, with these refinements, should be well aligned with the strength of the data and suitable for publication.*

**Response to editor:** We thank the Editor for the thoughtful and constructive feedback. In response, we have revised the manuscript to further temper the interpretation of the findings and align with the exploratory nature of the analysis. Specifically, we removed causal language and consistently framed the results as associative and hypothesis-generating. We also expanded the Discussion and Limitations to more clearly acknowledge key limitations, including the lack of adjustment for illness severity, potential residual confounding, small sample size, and imprecision of the estimates. In addition, we clarified the exploratory nature of the transcriptomic analysis, provided justification for the absence of multiple testing correction, and reframed IL7R as a candidate signal requiring further validation. Reverse causality has also been explicitly acknowledged. We believe these revisions have improved the clarity and rigor of the manuscript, and we appreciate the Editor’s guidance.

**Review Comments to the Author**

**Reviewer #1:** *This manuscript addresses an important and clinically relevant question regarding the impact of ICU-acquired infections on mortality in pneumonia-induced sepsis, while also exploring associated immune-related gene expression patterns using a publicly available dataset. The integration of clinical outcomes with transcriptomic data is a notable strength and adds value to the study. The revised version demonstrates improved clarity and a more appropriate acknowledgment of key limitations. The authors’ effort to refine the interpretation compared to earlier versions is appreciated. However, several important methodological constraints remain, and further refinement in the interpretation and framing of the findings is needed to ensure that the conclusions are proportionate to the strength of the data.*

Major Comments:
***1. Interpretation of mortality findings***
*The reported association between ICU-acquired infections and mortality, particularly in the CAP subgroup, is based on relatively small sample sizes and borderline statistical significance. In addition, the effect estimates are accompanied by wide confidence intervals, suggesting limited precision. While the interpretation has been moderated in the revised version, the conclusions may still be perceived as stronger than supported by the data.*
***Suggestion:*** *The authors are encouraged to further emphasize that these findings represent unadjusted associations and to explicitly acknowledge the uncertainty related to limited statistical power and imprecision of the estimates. Any causal language should be avoided, and the interpretation should remain appropriately cautious. In addition, the use of causal wording (e.g., “drive higher mortality”) in the title and conclusions should be reconsidered, as the study design supports association rather than causation.*

**Response to reviewer:** Thank you for this important and constructive comment. We have further revised the manuscript to ensure a more cautious and balanced interpretation of the mortality findings. Specifically, we have consistently removed any causal language and clarified that the observed relationship between ICU-acquired infections and mortality reflects associations rather than causation. We have also incorporated additional statements in the Discussion to explicitly acknowledge the observational nature of the analysis, as well as the potential influence of residual confounding, limited statistical power, and imprecision of the effect estimates. These points are now emphasized both in the interpretation of the findings and in the Limitations section. In addition, the title and conclusions have been revised to avoid causal wording.

***2. Lack of multivariable adjustment***
*The authors appropriately note that multivariable analysis was not feasible due to dataset limitations. However, this remains a critical limitation, as important confounding factors such as illness severity, ICU interventions, and comorbidities are not accounted for.*
***Suggestion:*** *This limitation should be more prominently emphasized in the Discussion. The authors should clearly state that the observed associations may be influenced by unmeasured confounding and should therefore be interpreted with caution.*

**Response to reviewer:** Thank you for this important comment. In response, we have performed multivariable logistic regression adjusting for available baseline variables (age, gender, and diabetes mellitus). We also explicitly acknowledged in the Discussion and Limitations sections that adjustment for illness severity and other potential confounders was not feasible due to dataset limitations. Accordingly, we emphasized that the observed associations may be influenced by residual confounding and should be interpreted with caution.

***3. Transcriptomic analysis and multiple testing*** *The manuscript appropriately describes the gene expression analysis as exploratory. However, the absence of correction for multiple comparisons should be more explicitly justified, as this increases the risk of false-positive findings.*
***Suggestion:*** *The authors should reinforce that these findings are hypothesis-generating and avoid implying confirmatory or clinically actionable conclusions. A brief justification for not applying multiple testing correction would improve transparency.*

**Response to reviewer:** Thank you for this important comment. We have revised the manuscript to further clarify the exploratory nature of the transcriptomic analysis. Specifically, we have emphasized in both the Methods and Discussion sections that the gene expression findings should be considered hypothesis-generating, given the focused candidate gene approach and the absence of formal correction for multiple comparisons. We have also added a brief justification for not applying multiple testing correction, noting that the analysis was limited to a small number of pre-specified candidate genes selected based on prior literature, rather than a genome-wide discovery approach. In addition, the interpretation of these findings has been further moderated to avoid any implication of confirmatory or clinically actionable conclusions.

***4. Interpretation of IL7R as a potential biomarker*** *The discussion suggests that IL7R may serve as a potential biomarker. Given the exploratory nature of the analysis and the absence of external validation or predictive performance assessment, this interpretation may be premature.*
***Suggestion:*** *The authors are encouraged to reframe IL7R as a candidate signal requiring further validation, rather than a clinically applicable biomarker at this stage.*

**Response to reviewer:** Thank you for this important comment. We have revised the manuscript to avoid overinterpretation of IL7R as a clinically applicable biomarker. Specifically, we have reframed IL7R as a candidate signal requiring further validation and moderated the language throughout the Discussion to reflect the exploratory nature of the analysis and the absence of external validation.

***5. Reverse causality*** *Gene expression was measured at ICU admission, while ICU-acquired infections occurred later during the ICU stay. Therefore, the temporal relationship between gene expression and infection risk remains uncertain.*
***Suggestion:*** *The Discussion would benefit from a more explicit consideration of potential reverse causality and a clear statement that causal inference cannot be established from the current analysis.*

**Response to reviewer:** Thank you for this important comment. We have revised the manuscript to more explicitly address the issue of reverse causality. Specifically, we clarified in the Discussion that the temporal relationship between gene expression and subsequent ICU-acquired infections remains uncertain, and that reverse causality cannot be excluded, limiting causal interpretation of the findings. We believe this addition appropriately reflects the limitations of the study design and avoids any implication of causal inference.

**Minor Comments:**
*1. Please ensure consistency in reporting mortality outcomes (28-day vs 30-day), as inconsistencies are currently present in the Abstract and Results sections.*

**Response to reviewer:** Thank you for this comment. We have reviewed the manuscript and ensured consistency in reporting mortality outcomes throughout. All instances have been standardized to 28-day mortality in both the Abstract and Results sections.

*2. The rationale for selecting the candidate genes should be clarified (e.g., whether they were pre-specified based on prior evidence).*

**Response to reviewer:** Thank you for this comment. We have clarified in the Methods section that the candidate genes were pre-specified based on prior evidence from the literature, focusing on genes with established roles in immune regulation and sepsis-related host response.

*3. Consider reporting effect sizes (e.g., fold changes) alongside p-values for gene expression results to improve interpretability.*

**Response to reviewer:** Thank you for this helpful suggestion. We have now addressed this concern by including fold change values for the selected genes (Table 4), which improves the interpretability of the gene expression results.

*4. Minor language editing is recommended to improve clarity and readability.*

**Response to reviewer:** Thank you for this suggestion. The manuscript has been carefully reviewed and revised to improve clarity, readability, and overall language quality.

*Overall, the manuscript has merit and addresses a relevant clinical question. With further refinement in interpretation and clearer positioning of the findings as exploratory and hypothesis-generating, it would be suitable for publication after minor revisions.*

***Reviewer #2:*** *Thank you for the revised manuscript. The authors have made several meaningful improvements, particularly in clarifying the methodology, providing effect size estimates, and moderating the interpretation of the findings. These revisions have strengthened the manuscript and addressed several of the concerns raised in the previous round. However, a few important issues remain.*

*1. The absence of any adjusted analysis continues to be a key limitation. Even without formal severity indices, adjustment for available baseline variables such as age, sex, and diabetes would still be feasible and would help clarify whether ICU-acquired infection is an independent predictor of mortality or simply a marker of more severe illness.*

**Response to reviewer:** Thank you for this important comment. In response, we have performed multivariable logistic regression analyses adjusting for available baseline variables, including age, gender, and diabetes mellitus. The results of both unadjusted and adjusted models are now presented in the revised manuscript (Table 3).

*2. In addition, the small sample size, particularly in the subgroup of CAP patients with ICU-acquired infection, substantially limits statistical power, and the borderline findings should be interpreted with caution.*
**Response to reviewer:** Thank you for this important comment. We have revised the manuscript to more clearly acknowledge the impact of the small sample size, particularly in the CAP subgroup with ICU-acquired infections. Specifically, we emphasized in the Discussion and Limitations sections that the findings are based on a limited number of cases, resulting in reduced statistical power and imprecision of the estimates, and should therefore be interpreted with caution. We have also moderated the language throughout the manuscript to reflect this uncertainty.
*3. While the transcriptomic analysis is now described as exploratory, the potential impact of multiple testing and the associated risk of false-positive findings could be more explicitly discussed. The rationale for selecting the specific candidate genes would also benefit from further clarification, particularly regarding whether this approach was hypothesis-driven.*

**Response to reviewer:** Thank you for this valuable comment. We have further clarified the exploratory nature of the transcriptomic analysis in both the Methods and Discussion sections. Specifically, we have emphasized that the findings should be considered hypothesis-generating, given the absence of formal correction for multiple comparisons and the associated risk of false-positive findings. In addition, we have clarified that the selected genes were pre-specified based on prior evidence from the literature, reflecting a hypothesis-driven candidate gene approach rather than a genome-wide discovery analysis. These revisions improve the transparency and interpretation of the transcriptomic findings.

*Overall, the manuscript has improved and addresses a clinically relevant question, but further clarification and more cautious interpretation of the findings would strengthen the validity of the conclusions.*
